# The Relationship Between Personal Values and Leisure‐Time Exercise: A Three‐Wave Study

**DOI:** 10.1002/pchj.70012

**Published:** 2025-03-29

**Authors:** Guozhuang Chen, Jiamei Liang, Chun Xie, Kun Wang

**Affiliations:** ^1^ Department of Physical Education Shanghai Jiaotong University Shanghai China; ^2^ School of Education Shanghai Jiaotong University Shanghai China

**Keywords:** exercise intention, exercise motivation, leisure‐time exercise, personal values

## Abstract

To gain more insight into why adolescents exercise, based on Schwartz's personal values model and self‐determination theory, this study examined whether personal values affect leisure‐time exercise behavior and their underlying mechanisms. Five hundred twenty‐two participants (193 Females and 329 Males; *M*_age_ = 19.32, SD = 1.02) in China were included. Structural equation modeling was used to examine the mediating and moderating effect. The results indicated that security‐personal can predict leisure‐time exercise, identified regulation, and exercise intention can mediate this relationship. Achievement can predict leisure‐time exercise, introjected regulation and exercise intention can mediate this relationship, and emotional social support for exercise can moderate the relationship between achievement and introjected motivation. Hedonism can predict leisure‐time exercise, intrinsic motivation and exercise intention can mediate this relationship. Conformity‐interpersonal is not related to leisure‐time exercise.

## 1 Introduction

Although extensive research has firmly established the psychological and physiological benefits of exercise (Bean and Forneris 2016; Young 2018; Penedo and Dahn 2005), a significant proportion of adolescents continue to engage in low levels of exercise (Tremblay et al. 2016; Agbo et al. 2018). In the past years, in order to motive adolescents to exercise, a great deal of motivational factors both in individual and environmental aspects have been prompted (Forrest et al. 2016; MacCann et al. 2015; Sylvester et al. 2018; Kaushal et al. 2021; Rhodes and Kates 2015), and some theories have been developed within the psychology literature (Biddle and Nigg 2000). Self‐determination theory, explained the mechanism of developing exercise decision from the perspective of behavioral drivers, has been mostly applied to illustrate the process of how an individual's exercise behavior occurs (Edmunds et al. 2006; Weman‐Josefsson et al. 2015). However, an individual's exercise motivation is influenced by various factors, it cannot provide an understanding of an individual's exercise behaviors at the level of core beliefs and life goals (Sagiv and Schwartz 2022). As a result, it can only drive individuals to exercise for a short period of time, rather than turning exercise into behaviors guided by an individual's core beliefs. This is not conducive to completely solving the current problem of individuals' insufficient exercise behavior. In order to explore factors that can drive individuals to participate in exercise at the belief level, this study, grounded in the self‐determined theory and Schwartz's personal values model, begins by focusing on a more stable and trans‐situational behavioral determinant—personal values—to provide a deeper understanding of the mechanisms underlying exercise behavior.

Personal values, a central aspect of the self‐concept, are defined as abstract beliefs that guide people's lives and are of significant causal importance as they shape people's perceptions, thoughts, and behaviors (Sagiv and Schwartz 2022; Hitlin 2003; Roccas and Sagiv 2017). Schwartz's personal values model, as the most widely used model in academic research (Rohan 2000; Maio 2010), constructs the structure of personal values based on the compatibility and conflict of the underlying motives they represent, has been validated in many countries (Schwartz 1992). This model classifies values into ten distinct types, which are further grouped into four overarching categories: self‐transcendence, self‐enhancement, conservation, and openness to change, with opposite values being mutually exclusive and neighboring values being similar (Schwartz 1992). Recently, some scholars have refined its classification to 19 categories, which allows for a more nuanced exploration of the relationship between personal values and behaviors (Schwartz et al. 2012; Schwartz 2017). While the pursuit of one personal value may conflict with opposing values, this does not imply that actions are driven by a single value alone. Instead, personal values are a motivational continuum, and each action is the result of multiple values working together (Roccas and Sagiv 2017).

Unlike other variables influencing an individual's life, personal values have three unique characteristics. Firstly, personal values are desirable. For example, people may be aware of their personality traits and become dissatisfied with them, but individuals are always satisfied with their personal values structure (Roccas and Sagiv 2017; Sagiv and Roccas 2021). Secondly, values serve as standards or criteria for making choices and generating behavior, the more important a value is, the more likely it is that people will act in a way that expresses that personal value (Schwartz 2017). Thirdly, although personal values are not constant, an individual's personal value structure is particularly stable and can be maintained over time (Roccas and Sagiv 2017; Bardi and Goodwin 2011). Therefore, as long as the person does not go through a significant event, the structure of personal values is constant over an extended period of time. In longitudinal studies of values‐behavior relationships, the requirements for a 6‐month time period for longitudinal studies can be shortened (Collins 2006). There are several caveats for the measurement of personal values: firstly, long questionnaires are preferable to short questionnaires; secondly, because values represent supervisory motivational tendencies, it is more appropriate to measure them using a self‐reported questionnaire (Roccas and Sagiv 2017). As a result, the Revised Portrait Value Questionnaire (PVQ‐RR) was used to measure personal values in this study.

As a proven psychological predictor for behaviors, personal value has a significant association with exercise. Honka et al. (2019) included Finnish Citizens as participants and indicated that conformity, power, security, achievement, benevolence, and universalism positively predicted physical activity levels, while hedonism was not related to physical activity. Worsley et al. (2013) indicated that universalism could positively predict the variance in physical activity habits, while conformity was not related to physical activity habits in Australian populations. Another research team indicated that both security‐personal and conformity‐interpersonal were negatively related to physical activity, while stimulation was positively related to physical activity among the Polish samples (Skimina et al. 2019, 2021). One relevant study in China and France proposed that hedonism positively predicted physical activity, while security‐societal, security‐personal, and conformity‐rules values negatively predicted physical activity, and achievement is not associated with physical activity (Liang et al. 2024). From the above studies, it can be concluded that the results of current research on the relationship between personal values and exercise behavior are inconsistent. Specifically, the inconsistencies mainly exist in those six values: security‐personal, security‐societal, hedonism, conformity‐interpersonal, conformity‐rules, and achievement. Therefore, further research is needed on the relationship between the above six categories of values and physical activity.

Hedonism, achievement, security‐personal, security‐societal, conformity‐rules, and conformity‐interpersonal are six of the 19 categories of personal values (Schwartz et al. 2012; Schwartz 2017). People with a high level of hedonism prefer to pursue pleasure and sensuous gratification for themselves; people with a high level of achievement prefer to pursue personal success through demonstrating competence according to social standards; people with a high level of security‐personal prefer to pursue a state of safety; people with a high level of security‐societal prefer to pursue a state of social stability; people with a high level of conformity‐rules prefer to compliance with laws and principles; people with a high level of conformity‐interpersonal prefer to avoid upsetting or harming other people (Schwartz et al. 2012; Schwartz 2017). From the above definition of values, it can be seen that the two values of security‐societal and conformity‐rules are less likely to drive individuals to exercise. This study therefore focuses on the relationship between the other four types of personal values and exercise behaviors.

Although both personal values and motivations serve as drivers of behavior, they differ fundamentally in several key aspects. Firstly, motivations are typically cyclical, whereas personal values are long‐term; secondly, personal values are cognitive representations, which people can easily bring to mind, allowing them to reflect on and consciously choose to pursue or ignore in certain situations, whereas motivations may not be consciously recognized; thirdly, personal values are transversal to situations and scenarios, whereas motivations are context‐dependent (Sagiv and Schwartz 2022). It has been argued that personal values do not directly influence behavior in the same manner as motivation does (Feather 1995), but rather, individuals are motivated to act in ways that express their personal values (Feather 1995; Bardi and Schwartz 2003). One study also indicates that some behaviors are value‐expressive, meaning they align with specific motivations and are thus consistently associated with the values that drive those motivations (Lönnqvist et al. 2013). As a result, it can be hypothesized that values may influence exercise behavior by shaping the exercise motivation associated with them.

Self‐determination theory posits that an individual's exercise behavior is driven by exercise motivation, which is categorized into two types: autonomous and controlled motivation. Autonomous motivation includes intrinsic motivation, integrated regulation, and identified regulation, while controlled motivation encompasses introjected regulation and external regulation (Deci and Ryan 2012). Identified regulation refers to the individual's recognition of the importance of engaging in exercise; introjected regulation motivates exercise behavior to avoid negative emotions; external regulation drives exercise behavior through the desire to meet external demands; and intrinsic motivation propels exercise behavior based on the individual's personal interests or passions (Deci 2002; Deci and Ryan 2012). Based on those definitions of motivation, it can be hypothesized that there may be a relationship between the four types of exercise motivation and the four categories of personal values within the 19 classifications: security‐personal, achievement, conformity‐interpersonal, and hedonism. Firstly, individuals with higher levels of security‐personal values are more focused on their own safety (Schwartz et al. 2012), which may lead them to place greater emphasis on their health and consequently pay more attention to exercise, resulting in higher levels of identified regulation. Secondly, individuals with higher achievement values often seek to demonstrate their abilities according to external standards (Schwartz et al. 2012). Since active participation in exercise is considered a marker of achievement, this group may be driven to exercise by higher levels of introjected regulation. Thirdly, individuals with higher hedonism values tend to seek personal pleasure and may therefore be motivated to engage in exercise due to their genuine interest in the activity, resulting in high levels of intrinsic motivation (Schwartz et al. 2012). Eventually, individuals with higher conformity‐interpersonal values are more concerned with the feelings of others and with not letting others down (Schwartz et al. 2012). As a result, they are likely to engage in exercise in response to encouragement from friends or family, resulting in high levels of external motivation. In addition, a substantial body of literature has investigated the relationship between various forms of exercise motivation and exercise behavior over the past years. Among them, a systematic review of 66 studies revealed that 91% of the research identified a significant positive correlation between intrinsic motivation, identified regulation, and exercise behavior; 30% of the studies reported a significant positive correlation between introjected regulation and exercise behavior; and nearly half of the studies demonstrated a significant negative or no correlation between external regulation and exercise behavior (Teixeira et al. 2012). Therefore, it is believed that the above four types of personal values may have an impact on individual exercise behavior through the four types of exercise motivations associated with them.

Intentions are explicit decisions to act in a certain way, and they express a person's motivation towards a goal (Sheeran 2002). One study pointed that intention is the most immediate and best predictor of behavior (Ajzen 1991), and some individuals develop an intention for a certain behavior, but when the expression of that intention fails, the actual behavior does not occur (Sniehotta et al. 2005). After an individual's motivation for a behavior arises, it may have an impact on behavioral intentions and thus influence behaviors. As a result, intentions maybe a mediator in the relationship between motivation and behaviors. In the field of sports, it has been suggested that an individual's exercise motivation can sometimes influence exercise intention, thereby affecting exercise behavior (Willem et al. 2017). However, in some cases, exercise motivation may only strengthen exercise intentions without actually leading to exercise behavior (Stanley et al. 2012). Obviously, exercise intention is a mediating variable in the way that exercise motivation influence exercise behavior. The stronger an individual's motivation to exercise, the more it reinforces exercise intention, thereby increasing the likelihood of developing an exercise routine that will encourage consistent exercise behavior. As a result, this study will not only explore the mediating role of exercise motivation in the relationship between personal values and exercise behaviors, but also the chained mediating role of exercise motivation and exercise intention in this relationship.

Although some scholars define personal values as a motivational variable, personal values do not directly influence behavior in the same way that motivation does, but are only one factor that individuals may consider when making conscious decisions (Schwartz 2017). There may be moderating factors between personal values and individual motivation, which are mostly derived from the socio‐cultural context in which the individual is situated (Schwartz 2017). The “value‐behavior” model also indicates that personal values can be equated with motivation to guide behavior in some cases and not in others, and socio‐cultural factors can moderate the relationship between personal values and behavior (Sagiv and Roccas 2021). For example, some scholars have found a significant moderating role of the socio‐cultural context in the value‐behavior relationship in religious activities (Yesilada et al. 2009). In the field of sport, exercise social support refers to encouragement, modeling, or other factors that arouse exercise behavior provided by individuals in a person's social network, and can be categorized into instrumental, emotional, informational, peer, and confirmatory support (Sallis et al. 1987; Zhong Tao and Liang 2019). Research has concluded that emotional exercise social support could enhance an individual's sense of identity and belonging in exercise participation, providing a positive cultural climate for an individual's exercise behaviors (Zhong Tao and Liang 2019). Therefore, as a cultural contextual factor, emotional exercise social support may be a moderator in the relationship between personal values and exercise motivation.

**FIGURE 1:**
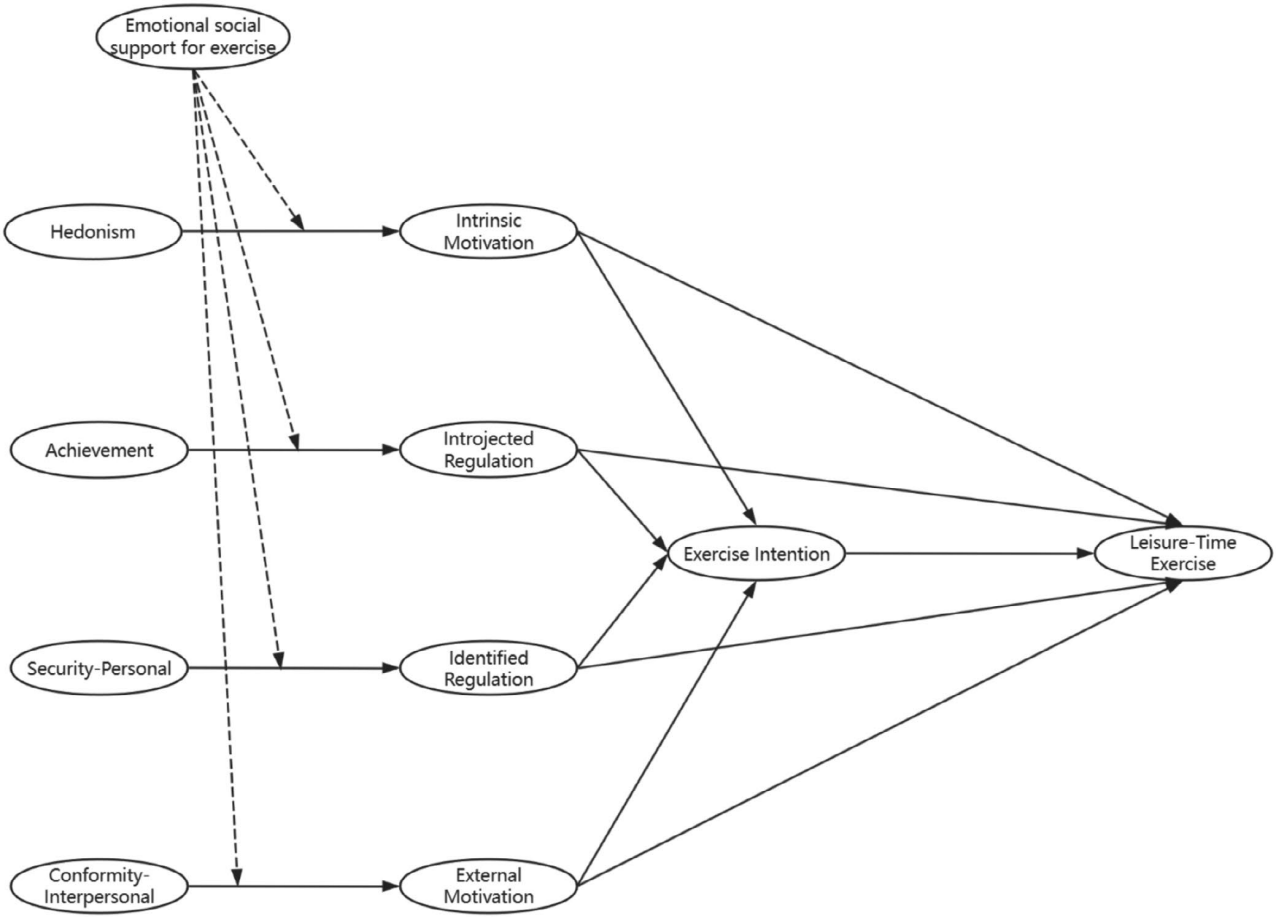
The theoretical model of personal values‐exercise behaviors (solid lines indicate normal impact and dashed lines indicate moderated impacts).

Based on the above discussion, we can find that current studies that explore the relationship between personal values and exercise behavior has the following shortcomings: firstly, current findings about the relationships between some personal values and exercise behaviors are inconsistent and require in‐depth research; secondly, longitudinal studies exploring the relationship between personal values and exercise behaviors are lacking; lastly, current research has not explored in depth the underlying mechanisms by which values influence individual exercise behaviors (e.g., exploration of mediating and moderating variables). As a result, the purpose of this study was to examine the effects of four personal values (hedonism, achievement, security‐personal, and conformity‐interpersonal) on leisure‐time exercise, meanwhile exploring the chain mediating role of exercise motivation and exercise intention in this relationship, and the moderating role of emotional social support of exercise between personal values and exercise motivation. The hypothesized model of this study was constructed (see Figure 1), and the research hypothesis is stated as follows. Hypothesis 1: hedonism can influence exercise behavior both through the individual mediating effect of intrinsic motivation and through the chain mediating effects of intrinsic motivation and exercise intention. Hypothesis 2: achievement can influence exercise behavior both through the individual mediating effect of introjected regulation and through the chain mediating effects of introjected regulation and exercise intention. Hypothesis 3: security‐personal can influence exercise behavior both through the individual mediating effect of identified regulation and through the chain mediating effects of identified regulation and exercise intention. Hypothesis 4: conformity‐interpersonal can influence exercise behavior both through the individual mediating effect of external motivation, and through the chain mediating effects of external motivation and exercise intention. Hypothesis 5: emotional exercise social support can moderate the relationships between four personal values and four exercise motivations (hedonism‐intrinsic motivation, achievement‐introjected regulation, security‐personal‐identified regulation, conformity‐interpersonal‐external motivation).

## 2 Materials and Methods

### 2.1 Participants

Five hundred twenty‐two college students with an average age of 19.32 years (SD = 1.02) from Shanghai Jiaotong University are included in this study. Among them, 329 (63%) were male, and 193 (37%) were female. The exercise types students reported included archery (6.1%), baseball (3.4%), basketball (60%), Tai Chi (5.2%), badminton (5.4%), table tennis (9.6%), swimming (3.4%), and cross‐country orienteering(6.9%). G*Power software was used to compute the minimum sample size that satisfies the condition, a linear multiple regression: fixed model test (power = 0.95, *α* = 0.05, within‐between interaction, effect size = 0.15) showed that a sample size of at least 107 valid subjects was required for this study. This indicates that the amount of valid data collected in this study is statistically efficient. This study was approved by the Ethics Committee at the Shanghai Jiaotong University (Approval Number: H20230292I).

### 2.2 Measurements

#### 2.2.1 Personal Values

The Revised Portrait Value Questionnaire (PVQ‐RR) was used to measure personal values in this study. The PVQ‐RR assesses 19 values, each with three items, totaling 57 questions (Schwartz et al. 2012). Examples of items on the PVQ‐RR scale is as follows: “It is important to him to take care of people he is close to.” The scales used a 6‐point Likert‐type scale, ranging from “1 = *very unlike me*” to “6 = *very like me*”. The higher the scores, the more important the value to participant. The Chinese version of this scale, validated in China, was utilized in this study (Schwartz and Cieciuch 2022). The Cronbach's alpha coefficient for the scale in this study was 0.93.

#### 2.2.2 Exercise Motivation

The Behavioral Regulation in Exercise Questionnaire‐2 (BREQ‐2) developed by Markland and Tobin (2004) was adopted in this study. This scale consists of 18 items and measures five types of exercise motivation: amotivation, external regulation, introjected regulation, identified regulation, and intrinsic regulation. Examples of items on the BREQ‐2 scale is as follows: “I exercise because people say I should.” Responses are provided on a 5‐point Likert scale, ranging from 1 (*very inconsistent*) to 5 (*very consistent*). Higher scores indicate greater levels of exercise motivation. The Chinese version of this scale, validated in China, was utilized in this study (Liu et al. 2015; Luo et al. 2022). The Cronbach's alpha coefficient in this study is 0.77.

#### 2.2.3 Exercise Intention

The Exercise Intention Scale was employed to measure participants' intention to exercise (Ajzen 2006). This scale was developed in 2006 and is now widely used in research on exercise intentions. This scale consists of three questions and measure the subject's exercise intention in the next 2 weeks. Example of items is as follows: “I plan to exercise at least three times a week for at least 20 min in the next two weeks.” Responses are provided on a 7‐point Likert scale, ranging from 0 (*very inconsistent*) to 6 (*very consistent*). Higher scores indicate greater levels of exercise intention. The Chinese version of this scale, validated in China, was utilized in this study (Fang 2012). The Cronbach's alpha coefficient in this study is 0.89.

#### 2.2.4 Exercise Behavior

The Godin Leisure‐Time Exercise Questionnaire was used to measure participants' exercise behavior (Godin and Shephard 1985). The questionnaire includes three questions, that were designed to find out how often participants engaged in vigorous, moderate, and low‐intensity exercise for at least 30 min a week on average during the previous 2 weeks. Items example is as follows: “In the past two weeks, how many times per week, on average, have you engaged in strenuous exercise (heart pounding and sweating, e.g., running, soccer, basketball, etc.) for more than 30 min during your leisure time?” Subjects' exercise level was judged by energy consumption (9 MET for a high‐intensity exercise, 5 MET for a moderate‐intensity exercise, and 3 MET for a low‐intensity exercise). The total score of an individual's exercise behavior is represented by the total value of energy expenditure (MET), higher scores indicate greater levels of exercise behavior. The Chinese version of this scale, validated in China, was utilized in this study (Renfang 2018).

#### 2.2.5 Social Support for Exercise

The most widely used measurement of social support for exercise is the Social support for exercise scale (Sallis et al. 1987), but some studies have pointed out that this scale are not satisfactory, and the friend support factors in the scale can't be aggregated to one factor (Walker et al. 2019). As a result, this study used the six questions included in the Exercise Social Support Scale developed by Zhong Tao and Liang (2019) to measure the emotional exercise social support. Items example is as follows: “I was recognized for my active participation in the workout.” The higher the scores, the stronger the participants' emotional social support. Its validity has been verified by previous study in China and the Cronbach's alpha coefficient for the scale in this study was 0.95.

### 2.3 Procedures and Statistical Analysis

In this work, we distributed questionnaires to students in the classroom. Since our measurement of personal values is more specific (measuring values categorized in 19 with 57 items), there are too many items in the total questionnaire. In order to reduce the burden of participants and not interrupt normal classroom routines, we adopted a three‐wave design, which permitted clarity and contextualization of measures at each data collection point (assessments were arranged based on the temporal precedence of associations specified within the hypothesized model: T1 = personal values and exercise motivations, T2 = exercise intention, T3 = leisure‐time exercise behavior). This approach avoids some drawbacks of cross‐sectional designs in relationship studies. Since personal values are generally constant, this study proposes that individuals' personal value levels prior to the T1 are the same as those at the T1. As a result, this study measured personal values and exercise motivation simultaneously in the first round of measurement. After 5 weeks, participants' exercise intention was measured. 2 weeks later, participants' leisure‐time exercise was measured. The use of 5‐week intervals between the first data collections and the second date collections is consistent with past works (Kalajas‐Tilga et al. 2022). The use of 2‐week intervals between the second data collections and the third date collections is also consistent with past works (Blanchard et al. 2008; Conner et al. 2010).

After completing the informed consent, three rounds of paper‐based questionnaires were distributed to undergraduate students at Shanghai Jiaotong University. In the first round, we distributed 537 questionnaires, after deleting 15 invalid questionnaires, remaining 522 questionnaires; in the second round, a total of 567 questionnaires were issued, and 551 valid questionnaires left after 16 invalid questionnaires had been removed; in the third round, 546 questionnaires were distributed, and 540 valid questionnaires left after 6 invalid questionnaires had been removed. After matching the data according to the students' registration numbers, 522 college students are included in this study.

SPSS 23.0 (IBM Inc. Chicago, IL, USA) and Mplus 8.0 were used for data analysis. Firstly, the Cronbach's alpha coefficient was calculated to evaluate the reliability of each scale. Secondly, descriptive statistics were provided to portray the profiles of each variable. Thirdly, Pearson correlation was used to analyze the relationships among the variables. Finally, structural equation modeling (SEM) was conducted to examine the relationship between personal values and leisure‐time exercise and the underlying mechanisms.

## 3 Results

### 3.1 Descriptive Statistics and Correlation Analysis

Mean, the standard deviation of each variable was presented in Table 1. The Pearson correlation coefficient of each variable is presented in Table 2. The results indicate that hedonism was significantly positively correlated with intrinsic motivation (*r* = 0.151, *p* < 0.001), achievement was significantly positively correlated with introjected regulation (*r* = 0.173, *p* < 0.001), security personal was significantly positively correlated with identified regulation (*r* = 0.254, *p* < 0.001), conformity interpersonal was significantly positively correlated with external regulation (*r* = 0.138, *p* < 0.001), and only hedonism was positively correlated with leisure‐time exercise, other three personal values were not correlated with leisure‐time exercise behaviors. In addition, exercise Intention was significantly positively correlated with intrinsic motivation (*r* = 0.451, *p* < 0.001), introjected regulation (*r* = 0.346, *p* < 0.001), and identified regulation (r = 0.485, *p* < 0.001), while negatively correlated with external regulation (*r* = −0.111, *p* < 0.05). Exercise Intention was significantly positively correlated with leisure‐time exercise (*r* = 0.337, *p* < 0.001). Leisure‐time exercise was significantly positively correlated with intrinsic motivation (*r* = 0.243, *p* < 0.001), identified regulation (*r* = 0.242, *p* < 0.001), and introjected regulation (*r* = 0.226, *p* < 0.001), but was not correlated with external regulation (*r* = −0.042, *p* > 0.05).

**TABLE 1:** Descriptive statistics.

Variables	M	SD
Hedonism	4.20	0.71
Achievement	3.70	0.85
Security‐personal	4.14	0.65
Conformity‐interpersonal	3.42	0.98
Emotional social support for exercise	2.15	0.77
Amotivation	0.94	0.89
External regulation	1.18	0.76
Introjected regulation	1.86	0.87
Identified regulation	3.00	0.64
Intrinsic motivation	2.89	0.79
Exercise intention	4.01	1.42
Leisure‐time exercise	42.22	26.48

**TABLE 2:** Correlation analysis for the study variables.

	Intrinsic motivation	Introjected regulation	Identified regulation	External regulation	Exercise intention	Leisure‐time exercise
Hedonism	0.151**	—	—	—	0.151**	0.094*
Achievement	—	0.173**	—	—	0.190**	0.034
Security personal	—	—	0.254**	—	0.155**	0.001
Conformity interpersonal	—	—	—	0.138**	0.026	−0.005
Intrinsic motivation	1		—	—	—	0.243**
Introjected regulation	—	1	—	—	—	0.226**
Identified regulation	—	—	1	—	—	0.242**
External regulation	—	—	—	1	—	−0.042
Exercise intention	0.451**	0.346**	0.485**	−0.111*	1	0.337**
Leisure‐time exercise	0.243**	0.226**	0.242**	−0.042	0.337**	1

* *p* < 0.05.

** *p* < 0.01.

*** *p* < 0.001.

### 3.2 Testing of Mediation Model

To control for potential confounding factors, *t*‐tests and analysis of variance were conducted to examine the differences in demographic variables (gender, age, exercise types) across each variable. None of these differences were significant (*p* > 0.05), indicating that demographic variables were unlikely to influence the model.

**FIGURE 2:**
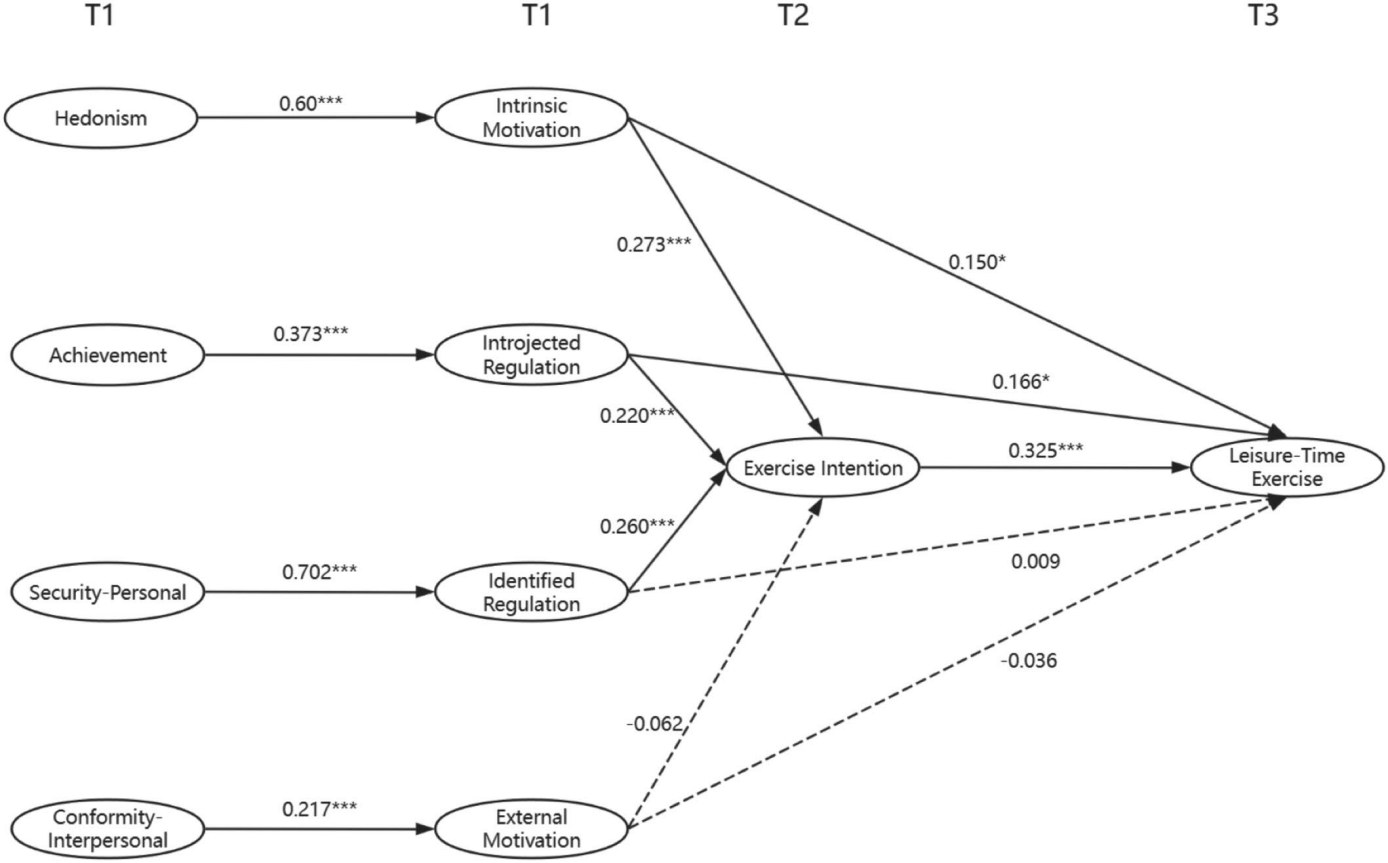
Mechanisms by which personal values influence exercise behavior (* *p* < 0.05, *** *p* < 0.001; solid lines represent significant impacts and dashed lines indicate insignificant impacts).

Afterwards, a mediating effect model was constructed to examine the relationship between the four personal values and leisure‐time exercise, and the results indicate a good model fit (RMSEA = 0.051, CFI = 0.910, TLI = 0.90, SRMR = 0.078). None of the four categories of personal values can influence exercise behaviors directly, but hedonism, achievement, and security‐personal can influence exercise behaviors indirectly. The standardized coefficients for all paths are shown in Figure 2. According to the results of the path analyses (Figure 2 and Table 3). Hedonism can positively predict intrinsic motivation (*β* = 0.6, *p* < 0.001), and influence exercise behavior by intrinsic motivation and exercise intention (*β* = 0.143, *p* < 0.001). Achievement can positively predict introjected regulation (*β* = 0.373, *p* < 0.001), and influence exercise behavior by introjected regulation and exercise intention (*β* = 0.089, *p* < 0.01). Security‐personal can positively predict identified regulation (*β* = 0.702, *p* < 0.001), and influence exercise behavior by identified regulation and exercise intention (*β* = 0.059, *p* < 0.01). Conformity‐interpersonal can positively predict external motivation (*β* = 0.217, *p* < 0.001), but can't influence exercise behaviors.

**TABLE 3:** Results of path analysis.

Path	Effect	Total effect
Hedonism → exercise intention → leisure‐time exercise	0.09***	0.143***
Hedonism → intrinsic motivation → exercise intention → leisure‐time exercise	0.053***	
Achievement → introjected regulation → leisure‐time exercise	0.062**	0.089**
Achievement → introjected regulation → exercise intention → leisure‐time exercise	0.027**	
Security‐personal → identified regulation → leisure‐time exercise	0.006	0.059***
Security‐personal → identified regulation → exercise intention → leisure‐time exercise	0.059***	
Conformity‐interpersonal → external motivation → leisure‐time exercise	−0.008	0
Conformity‐interpersonal → external motivation → exercise intention → leisure‐time exercise	−0.004	

** *p* < 0.01.

*** *p* < 0.001.

### 3.3 Testing of Moderation Model

**FIGURE 3:**
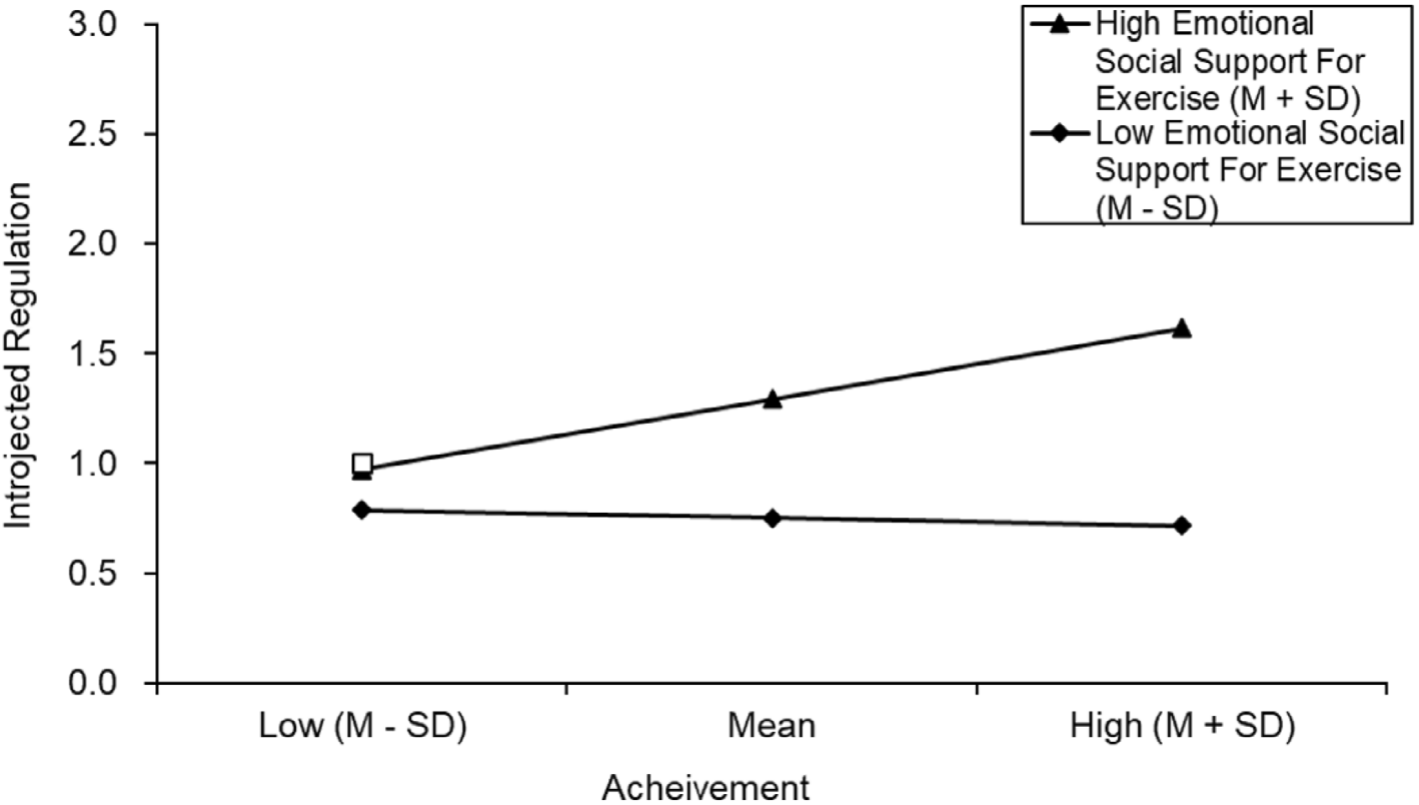
The moderating effect of emotional social support for exercise.

An examination of the moderating role of emotional exercise social support in the relationship between the four personal values and the four corresponding categories of exercise motivation found that emotional exercise social support can only moderate the relationship between achievement and introjected regulation. The enhancing effect of achievement on introjected regulation was more pronounced in individuals with higher emotional social support for exercise, and the interaction effect size was 0.258 (*p* < 0.001). For specific results see Table 4 and Figure 3.

**TABLE 4:** The coefficient of moderating effect.

Dependent variable	Independent variable and mediating variable	Estimate
Identified regulation	Security personal	0.245***
	Emotional social support for exercise	0.152***
	Security personal × emotional social support for exercise	−0.003
Intrinsic motivation	Hedonism	0.409***
	Emotional social support for exercise	0.401***
	Hedonism × emotional social support for exercise	−0.021
Introjected regulation	Achievement	0.169**
	Emotional social support for exercise	0.332***
	Achievement × emotional social support for exercise	0.258***
External regulation	Conformity‐interpersonal	0.208***
	Emotional social support for exercise	0.009
	Conformity‐interpersonal × emotional social support for exercise	−0.024

** *p* < 0.01.

*** *p* < 0.001.

## 4 Discussion

For the first time, we examined the relationship between four personal values and exercise behaviors in China, and investigated the underlying psychological mechanism in this longitudinal research. The results indicate that only hedonism is directly associated with leisure‐time exercise, while security‐personal, achievement, and conformity‐interpersonal do not have a direct relationship with leisure‐time exercise. This finding is not completely consistent with previous studies. Honka et al. (2019) suggested that security, achievement, and conformity positively predict physical activity levels, while Worsley et al. (2013) indicated that conformity is not related to physical activity habits. This discrepancy may be attributed to differences in the classification of personal values. For example, in Schwartz's model of personal values, security is divided into security‐personal and security‐social, which are distinct constructs. Consequently, it is expected that the relationship between these two variables and exercise behavior would differ (Schwartz 2017). Moreover, while most of the previous studies set physical activity level as a dependent variable, the present study focuses on investigating the relationship between personal values and leisure‐time exercise. Taking into account the difference between physical activity and leisure‐time exercise, it may be normal for the difference between those results. Physical activity is defined as bodily movement produced by skeletal muscle contraction, includes activities related to daily life, such as housekeeping, yardwork, occupational‐related, leisure‐related, and transportation (Fletcher et al. 2018). Exercise typically is differentiated from physical activity in that it is typically planned, repetitive, and structured with the main objective of improving health and fitness (Fletcher et al. 2018). Consequently, people's levels of physical activity and activity are not equal.

In addition, this study shows that some exercise motivations can mediate the relationship between personal values and leisure‐time exercise. Intrinsic motivation can mediate the relationship between hedonism and leisure‐time exercise, while introjected regulation can mediate the relationship between achievement and leisure‐time exercise. This implies that exercise motivation is a key factor in connecting these two personal values to exercise behavior, which is consistent with previous research findings. Compared to other behavior‐driving factors, personal values are broad, desirable goals that motivate actions and can influence preferences across different situations (Schwartz 1992). Consequently, personal values differ from constructs like attitudes, which pertain to specific actions or situations; personal values are a broader construct that guides behavior more generally (Sagiv et al. 2017). This implies that the direct predictive power of personal values for domain‐specific behaviors may be weaker or less pronounced compared to that of domain‐specific drivers of behavior. Exercise motivation, which reflects an individual's willingness to engage in exercise (Weman‐Josefsson et al. 2015), can translate broad, desirable goals associated with personal values into specific objectives within the sports domain, thereby directly influencing exercise behavior. For example, individuals with high levels of hedonism are more likely to engage in activities that bring them pleasure. However, this tendency towards seeking happiness may manifest in various behaviors that promote well‐being, rather than specifically driving engagement in exercise. Exercise motivation is an indication of hedonism’ increased accessibility in sports, thus can mediate the relationship between hedonism and exercise behaviors. As Oyserman (2015) and Verplanken and Holland (2002) state, the higher the accessibility of a value, the more likely it is to be activated and to directly influence behavior.

In contrast to the two previously discussed personal values, conformity‐interpersonal does not influence leisure‐time exercise through external regulation, which is consistent with prior research findings. As numerous studies have shown that external regulation is unrelated to exercise behaviors (Teixeira et al. 2012). Consequently, although conformity‐interpersonal can positively predict external regulation, it does not have a direct relationship with exercise behaviors. The results also indicate that identified regulation does not mediate the relationship between security‐personal and leisure‐time exercise. This finding is inconsistent with previous research, as numerous studies have demonstrated that identified regulation can directly and positively predict exercise‐related outcomes (Teixeira et al. 2012). This discrepancy may be attributed to the fact that this study was conducted longitudinally. Most studies demonstrating a significant relationship between identified regulation and exercise behavior have been cross‐sectional. However, some longitudinal studies have indicated no relationship between identified regulation and exercise behavior (Silva et al. 2010; Kwan et al. 2011).

This study further discovered that some personal values could predict leisure‐time exercise through the chained mediating role of exercise motivation and exercise intention. Specifically, exercise intention can mediate the relationship between autonomous motivations (intrinsic motivation and identified regulation) and leisure‐time exercise. This is consistent to previous research. As one meta‐analysis research indicates that self‐determined motivation can proceed intention and result in actual behavioral change (Hagger and Chatzisarantis 2009). This result implies that an individual's hedonism and security‐personal can influence leisure‐time exercise not only by enhancing autonomous motivation, but also by the chained mediating role of autonomous motivation and exercise intention. As a result, Hypothesis 1 and Hypothesis 3 have been proved.

In addition, we also found that exercise intention could mediate the relationship between introjected regulation and leisure‐time exercise, while this mediating effect was not appearing in the relationship between external regulation and exercise behavior, which is also consistent with previous studies.

As a result, Hypothesis 2 and Hypothesis 4 have not been completely proved. Many studies examining the relationship between exercise motivation and exercise intention indicate that introjected regulation is positively associated with exercise intentions in both adults and young people (Wilson and Rodgers 2004; Hagger et al. 2003). There is a study that reported the nonsignificant relationship between external regulation and exercise intention (Chatzisarantis et al. 1997). This implies that achievement can influence leisure‐time exercise behavior not only through introjected regulation, but through the chained mediating role of introjected regulation and exercise intention.

This study found that emotional social support for exercise significantly moderates the relationship between achievement and introjected regulation, suggesting that achievement can enhance exercise motivation to a greater extent in individuals with higher levels of emotional social support for exercise. This result is consistent with previous studies. The values‐behavior model indicates that culture can influence the instantiation of specific behavior and increase the likelihood that specific behaviors will be associated with relevant personal value (Sagiv and Roccas 2021). In other words, a supportive exercise environment helps individuals recognize that exercise behavior is strongly linked to achievement, thereby increasing their motivation to exercise. However, the moderating effect of emotional social support of exercise is not significant in the following three relationships: hedonism‐intrinsic motivation, security‐personal‐identified motivation, and conformity‐interpersonal‐external regulation. As a result, Hypothesis 5 has not been completely proved. The result suggests that emotional social support for exercise can only partially moderate the relationship between personal values and exercise motivation. This discrepancy may be attributed to two reasons below. Firstly, behavior is not guided by a single personal value alone, but by a whole system of personal values, each value has different priority status in the system (Roccas and Sagiv 2017). Relative to lower‐priority personal values, those higher‐priority personal values have a stronger relationship to behavior and are less susceptible to interference by external factors (Lake et al. 2024). In this study, the relationship between achievement and introjected regulation is weaker compared to the other three values‐behaviors relationships (see Table 4). As a result, the moderating role of emotional social support for exercise is more likely to show up in this relationship. Secondly, Elster and Gelfand (2021) points out that in a relatively relaxed and pleasant cultural context, individuals are more likely to express their personal values in their behaviors. As a result, both the strength and affinity of the cultural context have the potential to change the relationship between personal values and behaviors. Differences in cultural affinity may also account for the fact that moderation effects are not significant in some values‐behaviors relationships.

In summary, we conducted the first longitudinal study to examine the relationship between four categories of personal values and leisure‐time exercise behaviors, and elucidated the underlying psychological mechanisms. This study enriches the theoretical discourse on personal values and exercise behaviors, meanwhile provides practical insights for intervention strategies. Firstly, to date, this is the second study combining self‐determination theory and the personal values model to examine the mechanisms by which personal values influence exercise behavior. The first relevant study examined the mediating role of only two types of motivation (autonomous and controlled motivation) across four broad categories of personal values (self‐transcendence, self‐enhancement, openness to change, and conservation) and exercise behaviors based on cross‐sectional data (Liang et al. 2024). However, this approach fails to explore which specific types of values influence exercise behavior through which specific types of motivation. By focusing on more specific variables, we explored in greater depth the mechanisms by which personal values influence exercise behavior in a longitudinal study. Secondly, combined the Schwartz's personal values model and the self‐determined theory this study firstly investigated the moderating role of emotional social support of exercise in the relationship between personal values and exercise behaviors. This approach can deeper insights into how personal values affect exercise behaviors. In addition, since most current research on the determinants of exercise behavior focuses on short‐term predictors such as motivation, this study approaches the subject from the perspective of personal values—a psychological variable that remains relatively stable across time and situations. By doing so, it offers valuable insights into the factors that contribute to the long‐term formation of exercise habits in individuals.

The results of this study can provide policymakers or educators with several practical insights for increasing leisure‐time physical activity among youths. Firstly, considering the impact of different values on exercise behavior, in order to promote individual physical activity levels, educators can measure adolescents' personal values and design more individualized interventions based on their structural characteristics. Secondly, personal values‐based motivation is more enduring, so educational policymakers can address the lack of adherence to exercise among adolescents by focusing on fostering personal values that increase students' exercise motivation. Thirdly, since emotional social support of exercise can moderate the relationship between personal values and exercise motivation, educators can foster emotional social support of exercise among teenagers to raise their exercise motivation.

Several limitations of this study must be acknowledged. Firstly, from the perspective of exercise motivation, this study only examined the relationship between the four categories of personal values and exercise behavior based on the self‐determined theory. However, other personal values may also be associated with exercise behavior, as a result, future research can explore the relationship between other categories of personal values and exercise behavior. Secondly, although the data collection intervals in our study are supported by some literature (Kalajas‐Tilga et al. 2022; Blanchard et al. 2008; Conner et al. 2010), the intervals in this study still did not meet the 6‐month criterion, future research could add to this direction. Thirdly, although the sample size for this study was adequate, the samples were all from one university in China, the demographic homogeneity limits the generalizability. Therefore, in order to address this limitation, future studies could improve the generalizability of participants. Lastly, this study adopted a longitudinal research design, but different variables are measured at each time point, given the lack of auto‐regressive effects and potential confounds that could influence lagged associations. Future research can address this problem and adopt sophisticated analytic methods to validate the results of this study.

## 5 Conclusion

This study indicates that security‐personal can predict leisure‐time exercise, identified regulation, and exercise intention can mediate this relationship. Achievement can predict leisure‐time exercise, introjected regulation and exercise intention can mediate this relationship, and emotional social support for exercise can moderate the relationship between achievement and introjected motivation. Hedonism can predict leisure‐time exercise, intrinsic motivation, and exercise intention can mediate this relationship. Conformity‐interpersonal is not related to leisure‐time exercise.

## Ethics Statement

This study was approved by the ethics committee of the Shanghai Jiao Tong University (Approval number: H20230292I).

## Consent

Informed consent was obtained from all individual participants included in the study.

## Conflicts of Interest

The authors declare no conflicts of interest.
